# Steel Butterflies: Resilience and Positive Solitude Among Older Women in Israeli Kibbutzim During War

**DOI:** 10.1093/geroni/igaf122.386

**Published:** 2025-12-31

**Authors:** Sharon Ost-Mor, Lia Ring

**Affiliations:** University of Haifa, Haifa, HaZafon, Israel; Department of Social Work, Ruppin Academic Center, Emek Hefer, Israel

## Abstract

The October 7, 2023 attacks and subsequent war exposed women living in Israeli kibbutzim to collective trauma and forced evacuation, providing an opportunity to examine both psychological distress and potential growth in later life trauma exposure. This qualitative study examined the experiences of 27 women (ages 65-91) from three kibbutzim, including both evacuated and non-evacuated communities, using semi-structured interviews analyzed through thematic analysis to explore their experiences, coping mechanisms, and the role of positive solitude in trauma processing. Three primary temporal themes emerged: (1) “Wings Growing from Pain” - leveraging past experiences and personal strengths for present coping; (2) “Reconstruction Within Chaos” - creating new meaning while maintaining individual identity within the collective, particularly through positive solitary activities; and (3) “Tomorrow’s Garden” - future orientation expressed through both practical return plans and messages to younger generations, reflecting hope despite disappointment in institutional responses. The findings highlight the complex interplay between collective trauma and individual coping among women in kibbutzim. Their unique integration of resilience through positive solitude within a communal living framework demonstrates how life-course experiences contribute to trauma processing. This study offers insights for developing interventions that balance individual and collective needs in supporting people during crises, while acknowledging their capacity for growth even in the face of severe trauma.

